# Prevalence and clinical correlates of abnormal lipid metabolism in older Chinese patients with first-episode drug-naïve major depressive disorder

**DOI:** 10.1186/s12888-024-05967-x

**Published:** 2024-07-25

**Authors:** Xiao Huang, M. M. Yuan Sun, Xiang-Yang Zhang

**Affiliations:** 1 Department of Anesthesiology, Beijing Chao-Yang Hospital, Capital Medical University, No. 8 Workers’ Stadium South Road, Chaoyang Distinct, Beijing, 100020 China; 2 Department of Pharmacy, Peking University Third Hospital, No. 49 Huayuan North Road, Haidian District, Beijing, China; 3 Hefei Fourth People’s Hospital; Anhui Mental Health Center, 316 Huangshan Road, Hefei, 230022 China; 4 Affiliated Mental Health Center, Anhui Medical University, Hefei, China

**Keywords:** Major depressive disorder patients, Abnormal lipid metabolism, Prevalence, Clinical correlates

## Abstract

**Background:**

Older major depressive disorder (MDD) patients have more complex clinical symptoms and higher abnormal lipid metabolism (ALM) rates. This study aimed to compare clinical differences between those with and without ALM in a sample of older first-episode drug naïve (FEDN) patients.

**Methods:**

We recruited 266 older MDD patients. Socio-demographic variables, clinical data, and lipid parameters were obtained. The Hamilton Depression Rating Scale (HAMD), Hamilton Anxiety Rating Scale (HAMA), and the positive subscale of the Positive and Negative Syndrome Scale (PANSS-P) were conducted to evaluate patients’ depression, anxiety and psychotic symptoms, respectively.

**Results:**

In this study, we found that the prevalence of comorbid ALM was 86.1% in older MDD patients. Compared with the non-abnormal lipid metabolism (NALM) group, the ALM group had a higher duration of illness, higher clinical global impression of severity scale (CGI-S) and HAMD scores, higher thyroid stimulating hormone (TSH) and glucose levels. Logistic regression analysis indicated that duration of illness (OR = 1.11, *P* = 0.023, 95%CI = 1.015–1.216) and CGI-S score (OR = 2.28, *P* = 0.014, 95%CI = 1.18–4.39) were associated with ALM in older MDD patients.

**Conclusion:**

The importance of regular lipid assessment in older MDD patients needs to be taken into account.

## Introduction

Major depressive disorder (MDD) is a clinical condition characterized by low mood or feelings of sadness, loss of interest or pleasure in activities of daily living. Depressed patients often suffer from cognitive disturbances, low self-esteem, difficulty making decisions, feeling helplessness and despair. According to the World Health Organization (WHO), the lifetime prevalence of MDD is about 20% and will account for the primary contributor to disability worldwide by 2030 [1, 2]. In addition to conventional drug treatment, it is important for clinicians to understand the clinical characteristics of this population.

MDD is one of the most commonly observed mental disorders in older people and has a significant impact on both patients and caregivers. MDD in the elderly is usually underestimated and untreated because of its non-specific symptoms or is confused with other comorbidities such as heart disease, diabetes, malignancies, infections and major neurocognitive disorders. MDD is on the rise in the elderly population worldwide, with the global prevalence of MDD in elderly patients being 13.3% [3]. Sousa et al. demonstrated a prevalence of MDD of 47.3% in the elderly population, which was significantly higher than the prevalence of MDD in the general population (7.5–12.6%) [4]. Factors such as gender, living situation, mobility and nutritional status lead older people in different ways to social isolation and thus to loneliness, which then trigger MDD. Several studies have shown that MDD has a distinct symptom phenotype in older people compared to younger people. In China, middle-aged and older people (age ≥ 45 years) from the Health and Retirement Longitudinal Study with chronic or multiple illnesses are more likely to be depressed [5]. Common symptoms of MDD in older adults include emotional sadness, irritability, restlessness, insomnia, decreased appetite, psychomotor retardation, decreased energy, low self-esteem and suicidal thoughts [6]. In addition, older people are more frequently affected by severe cognitive impairments, which vary in severity and adversely influence functional ability [7]. Given concerns about potential negative effects on the older population, studying the clinical characteristics of older MDD patients is needed.

Lipids, which include sterols, di/triglycerides and phospholipids, are an integral component of biological membranes and are also involved in energy storage, production as well as cell signaling. Changes in lipids can influence the overall energy expenditure and basal metabolic rate owing to a sedentary lifestyle. Lipolysis of visceral fat promoted by adipose tissue lipoprotein lipase leads to excessive production of free fatty acids, resulting in insulin resistance and metabolic disease [8]. There is increasing evidence that depression is correlated with abnormal lipid metabolism (ALM) [9]. For example, Enko et al. found that depressed patients had higher TG and lower HDL cholesterol levels compared to healthy controls [10]. Lipidomics has been developed as a potential tool for detecting psychiatric disorders such as depression [11].

Serum lipid levels are mainly influenced by age, antipsychotics and antidepressants. Many studies have found a correlation between lipid metabolism and depression in older patients. For example, Vogelzangs et al. showed a general down-regulation of immunometabolism in elderly depressed patients, and the authors concluded that it is important to consider depressive symptoms when investigating dysbiosis in advanced-age depression [12]. Mulvahill et al. also found that the presence of metabolic syndrome (MetS) in elderly depressed adults was strongly correlated with the severity of depressive symptoms [13]. A prospective population-based 9-year study in Sweden found that apolipoprotein E (APOE) was associated with more severe symptoms of depression in advanced age, suggesting that APOE may identify people at high risk of clinically significant depression [14]. Lipids may increase the risk of depression and dementia in older adults by impairing the gabaergic signaling pathway and enhancing Glun2bglun2b phosphorylation [15]. However, another study found that neither APOE epsilon 4 nor epsilon 2 was associated with depression, late-onset depression, cognitive impairment, or psychomotor changes [16]. Since the relationship between lipid metabolism and elderly depressed patients has not been fully elucidated, studying the clinical correlates of ALM in older MDD is needed.

Recently, researchers have focused on depressive symptoms in older patients. However, the pathophysiological mechanisms behind ALM remain unclear. Although previous studies have revealed the relationship between lipid molecules and depression, there are still limited understandings of the role of ALM in older MDD patients. There are signs that dyslipidemia is correlated with the severity of depressive symptoms in the older, but it has not been researched in detail before. This investigation may shed light on the recognition of clinical characteristics for these two disorders. Therefore, it’s required for us to study the prevalence and correlates of lipid metabolism in elderly, first-episode, and drug naïve outpatients. This study aimed to assess the prevalence and clinical correlates of ALM in first-episode and drug-naive patients with MDD aged ≥ 50 years.

## Materials and methods

### Participants

Ethical approval was obtained from the Institutional Review Board of the First Hospital of Shanxi Medical University (No. 2016-Y27) and all methods were conducted in accordance with the Declaration of Helsinki, consistent with Good Clinical Practices and applicable regulatory requirements. Written informed consent was obtained from all participants before entering this study as well. We performed a cross-sectional study from 2016 to 2018 at the psychiatric outpatient department of the First Hospital of Shanxi Medical University, a general hospital in Taiyuan, Shanxi Province. Patients were asked by two independent psychiatrists in the way of a structured clinical interview on the Diagnostic and Statistical Manual, Fourth Edition (DSM IV) (SCID-I/P). The inclusion criteria were as follows: (1) Han Chinese patients aged ≥ 50 years old; (2) outpatients diagnosed with acute MDD by the criteria specified in the DSM IV at the start of the study; (3) had no history with antidepressants or antipsychotics and any other drugs; (4) Hamilton Depression Rating Scale (HAMD) score higher than 24; and (5) agreed to take part in the clinical assessment. The exclusion criteria were listed as follows: (1) being pregnant or breastfeeding; (2) drug abuse or alcohol dependence; (3) serious mental illness or personality disorder; (4) refusal to take part in the study; (5) difficulties with communication; (6) other unknown causes. Finally, a total of 266 patients were enrolled in the study.

### Sociodemographic data

Self-designed questionnaires including age, gender, height, weight, marital status, educational status, and duration of illness were used to obtain sociodemographic data. Trained nurses measured the blood pressure, height, and weight. We calculated body mass index (BMI) with the equation: BMI = Weight (kg)/Height (m)^2^.

### Clinical assessment

#### Depressive symptoms

The HAMD was used to evaluate the degree of depression. This scale consists of a 5-point scale (0 indicates not present, 4 indicates severe) and 9 3-point scales (0 indicates not present, 2 indicates severe). The presence and level of depression were determined by the total HAMD score [17].

#### Anxiety symptoms

The Hamilton Anxiety Scale (HAMA) was used to evaluate the anxiety degree of the participants. HAMA consists of 14 items with a 5-point Likert scale (0: absent, 4: severe), with the highest score of 56 points. The presence and level of anxiety were identified by the HAMA total score. Twenty-four was used as a cut-off value to diagnose severe anxiety symptoms [18].

#### Psychotic symptoms

The positive subscale of the Positive and Negative Syndrome Scale (PANSS-P) was used to evaluate patients’ psychotic symptoms with a total of 7 items each using a 7-point Likert scale (1 indicates not present and 7 indicates extremely severe). Fifteen was used as a cut-off value on the PANSS-P to diagnose MDD with psychotic symptoms [19].

Suicide attempts are defined as those who want to end their lives by some degree of self-harm [20]. We performed a self-designed questionnaire to evaluate suicide. “Have you ever in your life attempted to commit suicide?” If they answered in the affirmative, they were classified as suicide attempters. This was followed by further questions about the frequency, exact date and method of the suicide attempt. If the answer was inconclusive, we tried to find out more information by questioning their family and/or friends.

The clinical global impression of severity scale (CGI-S) was used to assess the severity of the disease. The CGI-S offers an assessment of current severity, with a score of 7 indicating the most severe case.

Two trained psychiatrists assessed the PANSS-P, HAMD, and HAMA scales before the study. For all the above scales, the correlation coefficient based on the two independent blinded raters was greater > 0.8.

### Measurement of thyroid function and metabolic parameters

Blood samples were obtained from each patient in fasting status between 6 am and 8 am and measured before 11 am for following biomarkers: cholesterol (TC), triglycerides (TG), high-density lipoprotein (HDL-C), low-density lipoprotein (LDL-C), fasting glucose, thyroid stimulating hormone (TSH), free thyroxine 3 (FT3), free thyroxine 4 (FT4), thyroid peroxidase antibody (TPOAb) and antithyroglobulin (TGAb). Then researcher sent all samples to the testing center of the First Hospital of Shanxi Medical University for analysis.

ALM is defined as one or more of the following: high TC as TC ≥ 200 mg/dl (5.20 mmol/L), or high TG as TG ≥ 150 mg/dl (1.70 mmol/L), or high LDL-C as LDL‐C ≥ 130 mg/dl (3.40 mmol/L), or low HDL‐C as HDL‐C ≤ 40 mg/dl (1.00 mmol/L) [21].

### Statistical analysis

According to the formula, n = Z^2^p(1-p)/d^2^ (n = number of samples; Z = 95% confidence interval equal to 1.96; d = 0.07 (7%), marginal error; p = expected prevalence, which we assumed to be 70%, based on previous study [22], an estimation of the sample of 182 patients was required, taking into account the elimination rate of patients. The sample size of this study was 266 cases, which was significantly larger than the required sample size, indicating that our sample size had sufficient validity.

SPSS version 26.0 (IBM, Chicago, IL, USA) was performed for analysis. The distribution of the sample was examined by Kolmogorov-Smirnov one-sample test. The chi-square test and one-way analysis of variance (ANOVA) were conducted for categorical and continuous factors, respectively. Mann Whitney *U* test was performed for non-normally distributed variables. Bonferroni correction was applied to adjust for multiple tests. Furthermore, logistic regression (Backward: Wald) was applied to detect the correlation of ALM and clinical and biochemical correlates of older MDD. Multicollinearity between independent factors was detected by variance inflation factors (VIF), with VIF > 5 indicating multicollinearity. In this study, two-tailed *P* values with a level of α = 0.05 were set as significant.

## Results

### Sociodemographic data between groups

A total of 266 older MDD patients were included in the final analysis, with 229 patients in the ALM group and 37 patients in the non-abnormal lipid metabolism (NALM) group. Of the 229 ALM patients, the majority (75.1%) were female, the mean age of the ALM group was 54.32 ± 3.38 years, 222 (96.9%) were married, and the mean duration of disease was 8.74 ± 6.07 months. Of the 37 NALM patients, the majority (70.3%) were female, the mean age of the NALM group was 54.16 ± 3.15 years, 35 (94.6%) patients were married, and the mean duration of disease was 5.26 ± 4.53 months.

### Clinical symptoms and metabolic indicators

There were no significant differences between the ALM group and the NALM group in terms of age, gender, marriage and education (all *P* > 0.05). But the duration of illness in ALM group was significantly longer than that in NALM group (*P* < 0.001). Table 1 presents the significant differences between ALM and NALM groups in terms of lipid and BMI levels, thyroid function parameters, HAMD, HAMA, psychotic symptoms, and suicide attempts. Compared with the NALM group, the ALM group had a higher level of TSH and fast blood glucose (all *P* ≤ 0.001). However, there was no significant difference in FT3 and FT4 between the two groups (all *P* > *0.05*). Furthermore, the ALM group had higher HAMD (F = 16.61, *P* < 0.001) and CGI-S (Z=-4.368, *P* < 0.001) scores. These variables survived after Bonferroni correction (*P < 0.05*).

**Table 1:** Demographic and clinical characteristics in older FEDN patients with MDD with and without ALM

	Without ALM (*n* = 37)	With ALM (*n* = 229)	F, Z or χ^2^	*P*
**Actual age**,** year**	54 (51,57.5)	54 (51,56.5)	-0.269	0.788
**Age of onset**,** year**	54 (51,57.5)	54 (51,56)	-0.681	0.496
**Duration of disease**,** month**	4 (3,6)	7 (4,13)	-3.794	< 0.001
**Sex**,** n (%)**			0.392	0.531
**Male**	11 (29.7)	57 (24.9)		
**Female**	26 (70.3)	172 (75.1)		
**Education**,** n (%)**			6.619	0.085
**Middle school**	20 (54.1)	139 (60.7)		
**High school**	11 (29.7)	74 (32.3)		
**College**	6 (16.2)	12 (5.2)		
**Graduate**	0	4 (1.7)		
**Married**,** n (%)**	35 (94.6)	222 (96.9)	0.537	0.463
**HAMD**	28.86 (3.28)	30.93 (2.78)	16.612	< 0.001
**HAMA**	20 (17,23)	21 (19,23)	-1.905	0.057
**PANSS-P**	7 (7,7)	7(7,10)	-1.982	0.05
**CGI-S**	5 (5,6)	6 (5,7)	-4.368	< 0.001
**TSH**,** mIU/L**	3.57 (1.87,4.66)	5.65 (3.89,7.4)	-4.956	< 0.001
**A-TG**,** IU/Ml**	21.16 (14.11,52.43)	22.3 (14.6,61.18)	-0.986	0.324
**A-TPO**,** IU/Ml**	17.23 (10.66,34.75)	18.68 (12.14,39.7)	-1.141	0.254
**Fating blood glucose**,** mmol/L**	5.03 (4.72,5.49)	5.45 (5.05,5.94)	-3.405	0.001
**HDL-C**,** mmol/L**	1.24 (1.16,1.6)	1.16 (0.96,1.37)	-3.21	0.001
**TG**,** mmol/L**	1.27 (1.13,1.53)	2.23 (1.63,2.86)	-7.404	< 0.001
**LDl-C**,** mmol/L**	2.43 (0.51)	3.16 (0.83)	27.6	< 0.001
**TC**,** mmol/L**	4.26 (0.63)	5.57 (1.02)	57.912	< 0.001
**FT3**,** pg/mL**	4.81 (0.69)	4.83 (0.76)	0.035	0.852
**FT4**,** ng/dL**	16.53 (3.26)	16.55 (3.06)	0.002	0.968
**BMI**,** kg/m**^**2**^	24.06 (1.53)	24.34 (1.98)	0.662	0.417
**Systolic blood pressure**,** mmgHg**	126.86 (7.74)	128.18 (8.76)	0.738	0.391
**Diastolic blood pressure(mmgHg)**	78.59 (8.2)	78.26 (8.93)	0.046	0.83
**Suicide**,** n (%)**	7 (18.9)	58 (25.3)	0.708	0.4
**Severe anxiety**,** n (%)**	5 (13.5)	38 (16.6)	0.223	0.637
**Exhibiting psychotic symptoms**,** n (%)**	3 (8.1)	39 (17)	1.907	0.167

Data expressed as mean ± SD, median (interquartile range), or percentage. HAMD: Hamilton Rating Scale for Depression. HAMA: Hamilton Anxiety Scale. PANSS-P: positive subscale of the Positive and Negative Symptom Scale. CGI-S: clinical global impression of severity scale. TSH: thyroid stimulating hormone. A-TG: anti-thyroglobulin. A-TPO thyroid peroxidases antibody. HDL-C: high-density lipoprotein cholesterol. TG: triacylglycerols. LDL-C: low-density lipoprotein cholesterol. TC: total cholesterol. FT3: free triiodothyronine. FT4: free thyroxine. BMI: body mass index

### Identifying ALM predictors

**Fig. 1:**
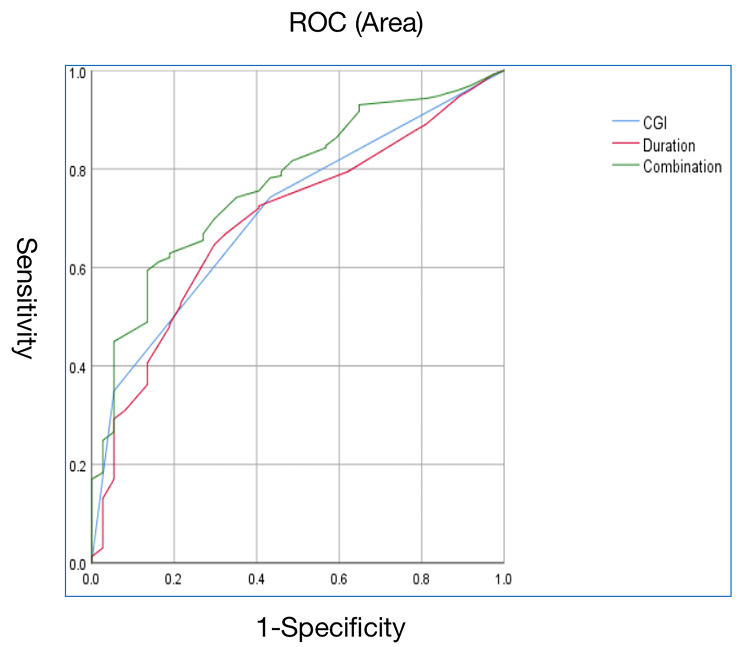
The discriminatory capacity of related factors for distinguishing between patients with and without ALM in older MDD. The area under the curve of CGI and duration of illness and the combination of these two factors were 0.71, 0.694 and 0.77 respectively. CGI: clinical global impression of severity scale. ALM: abnormal lipid metabolism. MDD: major depressive disorder

Binary logistic regression (Backward, Wald) was performed to identify predictors of ALM in older patients (see Table 2). Logistic regression analysis indicated that duration of illness (OR = 1.11, *P* = 0.023, 95%CI = 1.015–1.216) and CGI-S score (OR = 2.28, *P* = 0.014, 95%CI = 1.18–4.39) were associated with ALM in older MDD patients. The discriminatory capacity of related factors for distinguishing between patients with and without ALM in older MDD was presented in Fig. 1. The area under the curve of CGI and duration of illness and the combination of these two factors were 0.71, 0.694 and 0.77 respectively.

**Table 2:** The risk factors of ALM in older patients with MDD

	B	Wald	*P*	OR	95%CI Lower	95%CI Upper
**Duration of disease**	0.105	5.194	0.023	1.111	1.015	1.216
**CGI-S**	0.822	6.03	0.014	2.276	1.181	4.388
**TSH**	0.192	3.677	0.055	1.212	0.996	1.474
**Fating blood glucose**	0.605	3.072	0.08	1.832	0.931	3.605
**HAMD**	0.037	0.197	0.657	1.038	0.882	1.221

Note: CGI-S: clinical global impression of severity scale. TSH: thyroid stimulating hormone. HAMD: Hamilton Rating Scale for Depression

## Discussion

As far as we know, this was the first observational study in China to investigate the prevalence and clinical correlates of ALM in older patients with FEDN MDD. We found that the prevalence of ALM in older patients with FEDN MDD was as high as 86.1%. In addition, ALM was associated with longer disease duration, higher CGI and HAMD scores, and higher TSH and fasting blood glucose levels. Finally, we showed that higher CGI and longer disease duration were promising factors for assessing comorbid ALM in older patients with FEDN MDD.

We showed that the prevalence of ALM was 86.1% in this study. Consistent with our study, the study by Blank et al. also showed that abnormal metabolic values and MetS were very common in both younger and older patients with MDD: 87.6% of older (65–99 years) and 79.9% of younger patients had one or more metabolic abnormalities, 31.5% of older and 28.9% of younger patients had all metabolic abnormalities [23]. But De et al. found that the proportion of older adults aged 60 to 93 years with MDD, mood disorders, or mild depression who had MetS was 45.6% and 43.4%, respectively [24]. Macías-Cortés et al. stated that among Mexican women (40–65 years) with depression, 52.3% had hypertriglyceridemia and 44.7% had hypercholesterolemia [25]. The prevalence of low HDL and elevated triglycerides in patients with bipolar disorder was 71.6% and 64.2%, respectively, and MetS was significantly associated with a longer duration of illness [26]. The above three studies reported a significantly lower incidence of ALM compared with the present study. These studies included patients with MDD aged 60 to 93 years or Mexican women (40–65 years) with depression, but we included patients aged > 50 years old. Also, MetS in the above study was defined according to the National Cholesterol Education Program Adult Treatment Panel III criteria or Triglycerides and cholesterol, but we defined ALM according to four lipoproteins [21]. All of these factors may contribute to the difference in the prevalence of ALM in older adults with MDD. In this study, we measured four lipid levels to elucidate clinically relevant features of ALM in patients with older MDD. The causes of ALM are complicated by the fact that elderly patients often have multisystem diseases and are on multiple medications such as anti-inflammatories and analgesics. Also, the result of direct sympathetic activation may be a pivotal item in the link between mood disorders and ALM [27]. Furthermore, dietary differences in the target population cannot be ignored. For example, dietary polyunsaturated fatty acids (PUFAs) have been identified to be associated with reduced TG levels [28].

It should be noted that longer disease duration is associated with ALM in older patients with FEDN MDD. Previous work has also found a strong correlation between disease duration and ALM. For example, Scola et al. showed that the ratio of fatty acid levels in patients with MDD was correlated with disease duration [29]. The association between longer disease duration and ALM in older MDD patients may be due to structural changes in the brain [30]. Furthermore, patients with a longer duration of illness may be more likely to take psychotropic, lipid-lowering and anti-inflammatory medication, which may affect blood lipid levels. These findings indicate that it is important to routinely assess the duration of illness in older MDD patients with comorbid ALM.

We found that TSH levels were significantly higher in older MDD patients with ALM than in patients without ALM. The thyroid hormones are closely linked to blood lipids due to their unique physiological functions. Walczak et al. showed that triiodothyronine can influence lipid remodeling in adipocytes via the hypothalamus and other central nervous systems and TSH may modulate lipid metabolism by regulating the production and breakdown of lipids [31]. Ingestion of high doses of T3 and T4 accelerates lipid utilization by surrounding tissues while accelerating glucose uptake and intrahepatic glucose production. Although no significant difference in FT3 and FT4 was found between older MDD patients with and without ALM in our study, we pointed out that TSH levels were higher in the ALM group than in the NALM group. Higher TSH levels may cause endothelial dysfunction, which in turn leads to ALM and related complications. Hypothyroidism or hyperthyroidism is directly correlated with the risk of clinical depression. Also, dysregulation of the hypothalamic-pituitary-adrenal (HPA) axis and the hypothalamic–pituitary–thyroid (HPT) axis in depressed patients are the causes of abnormalities in glucose metabolism and thyroid metabolism, respectively [32, 33]. TSH levels are closely correlated with the development and progression of autoimmune thyroid disease, but their specific effect on blood lipids needs to be further explored.

Our results also suggest a higher abnormal glucose level in older MDD with ALM compared with those without ALM. In agreement with our results, De et al. found that the fasting blood glucose levels in depressed men and women were 6.1 and 5.8 mmol/L, which were above the normal range [24]. Previous studies have shown that insulin action in the hypothalamus of patients with major depression is impaired with increased visceral adiposity and decreased lipocalcin levels, which then reduce insulin sensitivity in patients with major depression [34]. Zhang et al. has also found that diabetes is an independent risk factor for advanced post-stroke depression (PSD) [35]. Metabolic dysfunction may facilitate the development of deregulated neuronal activity in the critical limbic region of MDD patients [36]. Diabetes can cause endothelial cell dysfunction and impairment of the blood-brain barrier, eventually leading to cerebral white matter lesions and vascular dementia, which are closely associated with the development of depression. Depressed patients with ALM are more likely to have poor lifestyle habits and leads to more severe metabolic disturbances in the body. Therefore, a history of abnormal glucose levels in older MDD patients may predict a higher risk of developing ALM, and appropriate interventions are urgently needed in this patient group.

The present study has several limitations. Firstly, the cross-sectional design of the present study could not account for the causal relationship between ALM and its risk factors; a prospective cohort study is needed for further investigation. Secondly, as our sample only included outpatients, the results of this study cannot be generalized to other patients. Thirdly, to clarify the hypothesis that poor treatment of ALM may be accompanied by more problems, we compared the variables of interest for the group with and without an ALM diagnosis. Despite the adequacy of our sample size, there were only 37 patients in the NALM group, leading to the cautious interpretation of our results. Our study can only be considered as a preliminary study, and future multicenter, large-scale prospective studies are needed to further validate the results of this study. Fourthly, unfavorable lifestyle factors such as lacking exercise (confounding factors such as dietary practice, exercise and lifestyle, substance use, and psychotropic drugs) in older MDD patients can have a serious influence on lipid metabolism. The effect of drugs on lipid composition cannot be ruled out. It is widely recognized that antipsychotics can impact lipid metabolism. However, we didn’t explore the impact of diet and lifestyle on lipid metabolism in older MDD patients. These limitations will all be addressed in further study. Finally, since this study is a secondary analysis of a cross-sectional study, limited by the inclusion of the population at the beginning, we can only choose patients between 50 and 60 years old. Strangely, patients over the age of 60 were not included in the study. In this case, it cannot strictly represent elderly patients. Thus, the older MDD in this study was only for patients who were relatively older compared to younger patients (18–50 years old). Therefore, our study needs to be interpreted with caution.

## Conclusion

In this study, we found that the prevalence of comorbid ALM was 86.1% in older patients with FEDN MDD. In addition, older MDD patients with ALM had longer disease duration, higher HAMD and CGI-S scores, and significant differences in blood glucose and TSH levels, indicating the specificity of clinical correlates and metabolic markers for ALM in older patients with FEDN MDD. Considering the significant imbalance in the gender ratio, with more than 70% of the participants being female in this study, future research incorporating gender-specific analyses and exploring additional methodological improvements is required to enhance the robustness and applicability of the results and provide a reliable basis for broader generalizations.

## Data Availability

The datasets generated and/or analyzed during the current study are not publicly available due to confidentiality concerns but are available from the corresponding author on reasonable request.

## Declarations

### Copyright license agreement

The Copyright License Agreement has been signed by all authors before publication.

## Abbreviations

MDD: Major depressive disorder
WHO: World Health Organization
ALM: Abnormal lipid metabolism
DSM IV: Structured clinical interview on the Diagnostic and Statistical Manual, Fourth Edition
HAMA: Hamilton Anxiety Rating Scale
HAMD: Hamilton Depression Rating Scale
BMI: Body mass index
HAMA-14: 14-item Hamilton Anxiety Scale
PANSS-P: Positive subscale of the Positive and Negative Symptom Scale
CGI-S: Clinical global impression of severity scale
TC: Cholesterol
TG: Triglycerides
HDL-C: High-density lipoprotein
LDL-C: Low-density lipoprotein
TSH: Fasting glucose, thyroid stimulating hormone
FT3: Free thyroxine 3
FT4: Free thyroxine 4
TPOAb: Thyroid peroxidase antibody
TGAb: Antithyroglobulin
ANOVA: One-way analysis of variance
VIF: Variance inflation factors
MetS: Metabolic syndrome
